# Management of hepatitis B in pregnant women and infants: a multicentre audit from four London hospitals

**DOI:** 10.1186/1471-2393-13-222

**Published:** 2013-12-01

**Authors:** Gauri Godbole, Dianne Irish, Marina Basarab, Tabitha Mahungu, Andrew Fox-Lewis, Claire Thorne, Michael Jacobs, Geoffrey Dusheiko, William MC Rosenberg, Deepak Suri, Andrew D Millar, Eleni Nastouli

**Affiliations:** 1 Department of Clinical Microbiology and Virology, University College London Hospitals NHS Foundation Trust, London, UK; 2 Department of Virology, Royal Free London NHS Foundation Trust, London, UK; 3 Department of Hepatology, North Middlesex University Hospital NHS Trust, London, UK; 4 Department of Paediatric Epidemiology and Biostatistics, UCL Institute of Child Health, University College London, London, UK; 5 Department of Infectious Diseases, Royal Free London NHS Foundation Trust, London, UK; 6 Institute of Liver and Digestive Health, Division of Medicine, University College London, London, UK; 7 Department of Hepatology and Gastroenterology, Whittington Health NHS Trust, London, UK; 8 Department of Gastroenterology and Hepatology, University College London Hospitals NHS Foundation Trust, London, UK; 9 Department of Clinical Microbiology and Virology, University College London Hospitals NHS Foundation Trust, 60 Whitfield Street, London W1T 4EU, UK

**Keywords:** Infection, Pregnancy, Hepatitis B, Antivirals, High risk

## Abstract

**Background:**

Pregnant women with hepatitis B virus (HBV) infection can transmit the infection to their infants, screening of patients and appropriate interventions reduce vertical transmission. This audit was conducted to assess adherence to the national guidelines for management of HBV infection in pregnancy.

**Methods:**

A retrospective audit was conducted on pregnant women diagnosed with hepatitis B on screening in antenatal clinics, across four hospitals in London over 2 years (2009–2010). Data was collected from antenatal records and discharge summaries using a standard audit form. The outcomes measured included HBV serological markers, HBV DNA, detection of other blood borne viruses and referral to hepatology services, administration of active and passive prophylaxis to infants at birth. Descriptive statistics are presented. Proportions were compared using the *χ*^2^ test and 95% confidence intervals (CI) were calculated for prevalence estimates. Analyses were conducted using STATA 12.

**Results:**

HBsAg was detected in 1.05% (n = 401, 95% CI 0.95-1.16) of women attending an antenatal appointment, 12% (n = 48) of the women were at a high risk of vertical transmission (HBe Ag positive or antiHBe and HBeAg negative or HBV DNA >10^6^ IU/ml). Only 62% (n = 248) women were referred to hepatology or specialist clinics and 29% (n = 13) of women of high infectivity were on antiviral agents. Testing for hepatitis C and delta virus was suboptimal. 75% (n = 36) of the infants at a high risk of acquisition of HBV received both active and passive prophylaxis.

**Conclusion:**

In certain sectors of London, implementation of the pathway for management of women with hepatitis B and their infants is suboptimal. National guidelines should be followed and improved intersectorial sharing of information is needed to reduce the risk of women of high infectivity being lost to follow up.

## Background

Chronic hepatitis B virus (HBV) infection remains endemic in many parts of the world and there are over 2 billion infected individuals worldwide [1]. The risk of chronicity of hepatitis B is inversely related to the age of acquisition of infection; vertical transmission is associated with a risk of chronicity of more than 80% [2]. Infants with chronic infection have a 25% lifelong risk of developing cirrhosis and/or hepatocellular carcinoma [3]. Screening of pregnant women for HBV and managing infected women and their infants appropriately in order to interrupt mother-to-child transmission is therefore of paramount importance.

The risk of vertical transmission of HBV is 70–90% when the woman is hepatitis B e antigen (HBeAg) positive, and around 40% when HBeAg is absent [4-7]. Active or passive immunisation or both reduces the risk of vertical transmission by 90% [8]. A high maternal viral load (VL) increases risk of transmission to the child [9], with babies born to women who are HBeAg positive and have a high VL (above 2 × 10^7^ IU/ml or log^8^ copies/ml) having an estimated transmission risk of at least 10% despite use of hepatitis B immunoglobulin (HBIG) and vaccination [10]. Use of antivirals in pregnancy is suggested as a means to reduce this risk [10-12]. However safety and efficacy data on use of antivirals for transmission purposes are not robust.

The UK Department of Health first published guidelines for antenatal screening for hepatitis B, vaccination and HBIG prophylaxis for infants at high risk of transmission, contact tracing and referral pathways in 1998 [8]. These best practice guidelines were updated in April 2003 [13] and more recently in 2011 [14-16]. The NHS Infectious Diseases in Pregnancy Screening (IDPS) Programme in England is responsible for ensuring that all pregnant women are routinely offered screening for hepatitis B, together with HIV, syphilis and susceptibility to rubella as part of their antenatal care. Routine antenatal screening for hepatitis B for all pregnant women became national policy in April 2000. All women confirmed as hepatitis B surface antigen (HBsAg) positive, and those with a prior diagnosis, should have infectivity markers, i.e. HBeAg and antibody (anti-HBe) determined and be promptly referred to an appropriate specialist service. HBV vaccination is recommended for all exposed babies, with those born to mothers at high risk of transmission offered HBIG as well as vaccination [13]. In late 2008, HBV DNA was included in the risk assessment of transmission determining the need for HBIG administration at birth [14] and in September 2010, the IDPS Programme published updated standards for hepatitis B screening, including referral pathways to hepatology/specialists, and screening of family [15]. Overall there is high coverage of antenatal screening for hepatitis B in the UK; in London, 98% of pregnant women were screened in 2006, with approximately 1200 infants born to mothers with HBV infection annually [17]. In England, in 2011 0.42% of pregnant women screened for HBV were HBsAg positive overall, with the highest prevalence in London at 1.02%, this represented a slight decline in overall prevalence over the past five years, from 0.47% in 2007 [18].

Despite existence of national guidelines, we observed that practices varied with regards to clinical management of pregnant women with hepatitis B, and their infants, and therefore performed an audit with maternity, hepatology, virology and paediatric colleagues in North Central London to assess adherence to UK guidelines for management of pregnant women with hepatitis B and their infants neonates [13,14] in 2009 and 2010. A secondary objective was to estimate the prevalence of HBsAg seropositivity in pregnant women in this setting, and to describe the characteristics of women with chronic hepatitis B.

## Methods

An audit was carried out across four teaching and district general hospitals in London providing antenatal services to the local population of approximately seven boroughs of London following the observation of variable practices in clinical management of hepatitis B in pregnancy. The audit population was pregnant women booking for antenatal care from 1^st^ January 2009 to 31^st^ December 2010 with hepatitis B, i.e. those testing positive for HBsAg at booking or with a prior diagnosis. For the HBsAg prevalence estimates, data on all pregnant women booking during this time period were used.

Data were collected using an audit data form, and were retrospectively extracted from antenatal booking records, pharmacy records and maternal and neonatal discharge summaries. The audit was registered in all four centres and was compliant with local clinical governance policies. The management of HBsAg seropositive women and their neonates was audited against the national guidance/standards applicable in 2009 and 2010 (Table 1). Data on demographics (age at booking, ethnicity), HBV serological markers (Hepatitis B e antigen [HBeAg] and antibody to e antigen [anti HBe]), HBV DNA testing, confirmatory serology, other tests (delta virus, HCV, HIV), maternal antiviral therapy, maternal referral and neonatal immunisation (HBIG and/or vaccine) were collected.

**Table 1:** Audit standards used

**2003 Standards to support the UK antenatal screening programme [**[13]**]**		
• Antenatal screening for Hepatitis B should be offered to all women at booking		
• Infectivity markers HBeAg and anti HBe determined for all samples confirmed as HBsAg positive; other		
• Markers at discretion of physician		
• Initial clinical assessment of women identified as HBsAg positive is carried out at the earliest opportunity		
• By those with expertise in managing hepatitis B/hepatology		
• Referral of partner and family for screening		
• First dose of vaccine given at or shortly after birth.		
• Immunisation of infant as follows:		
	**Vaccine**	**HBIG**
HBsAg positive & HBeAg positive	Yes	Yes
HBsAg positive without e markers	Yes	Yes
Acute hepatitis B during pregnancy	Yes	Yes
HBsAg positive and anti-HBe positive	Yes	No
**2008 update to immunisation green book **[14]**: Immunisation of infant as above plus:**		
HBsAg positive, HBeAg negative, anti-HBe negative	Yes	Yes
HBsAg positive & known to have HBV DNA	Yes	Yes
>1 x 10^6^ IUs/ml in an antenatal sample*		
**2008 British viral hepatitis group guidelines **[22] • All newly diagnosed women should undergo appropriate testing, assessment and referral as for non-pregnant individuals (including HBV DNA, Delta virus, HCV, HIV		
• Women with HBV DNA >10^7^ IU/ml should be considered for therapy with a potent antiviral agent from 32 weeks of pregnancy		

*Where DNA measurement has been performed to inform maternal management. HBV (hepatitis B virus), HBsAg (hepatitis B surface antigen), HBeAg (hepatitis B e antigen), anti HBe (hepatitis B e antibody), HBIG (Hepatitis B immunoglobulin).

### Definitions

HBV DNA (viral load -VL) was quantified as log IU/ml, and categorized as >10^7^, 10^6^ to 10^7^, 10^2^ to 10^5^ IU/ml and undetectable.

### Statistical analysis

The data from the four centres were assimilated in a common database and analysed. For prevalence estimates, 95% confidence intervals (CI) were calculated. Descriptive statistics are presented. Proportions were compared using the *χ*^2^ test and 95% confidence intervals (CI) were calculated for prevalence estimates. Analyses were conducted using STATA 12 (Stata Corp, College Station, Texas, USA).

## Results

### Prevalence of HBsAg seropositivity and characteristics of pregnant women with HBV

**Figure 1:**
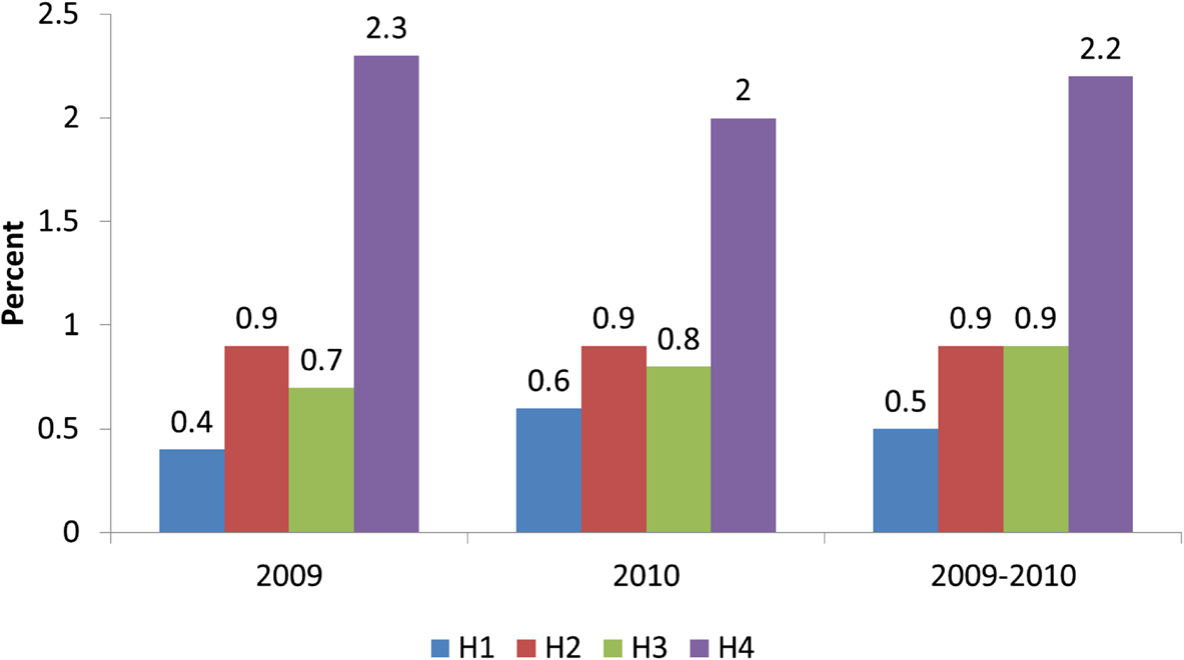
Prevalence of Hepatitis B (HBsAg positive) among women booking for antenatal care, by hospital of booking (H1,H2,H3,H4), 2009–2010.

A total of 38,227 pregnant women were booked for antenatal care during 2009 and 2010, of whom 401 were HBsAg positive (1.05%; 95% CI 0.95, 1.16) overall. There was no difference in prevalence between 2009 (194/18473, 1.05%) and 2010 (207/19755, 1.05%) (*p* = 0.5). Hospital 4 (H4) had significantly higher prevalence than the other three hospitals (*p* < 0.001), with prevalence of ≥2% in both 2009 and 2010 (Figure 1). There were no cases of acute hepatitis B noted during the study period.

Table 2 shows the socio-demographic characteristics of the pregnant women with hepatitis B by hospital: overall, 39% of women were black African (n = 158), 21% Asian (mainly Chinese) (n = 84), 19% white (n = 78), 17% (n = 67) other or mixed ethnicity, with 4% (n = 14) having no information available. Median age at antenatal booking was 29 years (range of 15 to 46 years). With respect to serological markers of infectivity, 9% (34/401) of women were HBeAg positive and anti HBe negative, 90% (359/401) were HBeAg negative, anti HBe positive and eight women were HBeAg negative and anti HBe negative. The proportion of women with high infectivity markers was similar across the study hospitals (Table 2). Sixty- six percent of the women across the 4 hospitals had their serology reconfirmed (263/401) (Table 2).

**Table 2:** Socio-demographic and serological markers of infectivity in pregnant women with hepatitis B, stratified by hospital (n = 401)

	**Hospital 1 (H1)**	**Hospital 2 (H2)**	**Hospital 3 (H3)**	**Hospital 4 (H4)**
	**Median (range) or N (%)**			
HBsAg positive	N = 70	N = 89	N = 64	N = 178
Age (years)	32 (18–44)	28 (17–45)	30 (20–43)	26 (15–46)
Ethnicity				
Black African	20 (29)	45 (51)	19 (30)	74 (42)
Asian	27 (39)	20 (22)	15 (23)	22 (12)
White	8 (11)	11 (12)	19 (30)	40 (22)
Other/mixed	12 (17)	7 (8)	9 (14)	39 (22)
Not stated	3 (4)	6 (7)	2 (3)	3 (2)
HBV serology				
HBeAg positive and anti HBe negative	8 (11)	7 (8)	5 (8)	14 (8)
HBeAg negative and anti HBe positive	60 (86)	82 (92)	58 (91)	159 (89)
HBeAg negative and anti HBe negative	2 (3)	0	1 (2)	5 (3)
Confirmatory HBV serology	59 (84)	72 (81)	27 (42)	105 (59)

HBV (hepatitis B virus), HBsAg (hepatitis B surface antigen), HBeAg (hepatitis B e antigen), anti HBe (hepatitis B e antibody).

### Management of pregnant women with HBV infection

In all four hospitals, referrals to hepatology services were made via Maternity by the Specialist Midwife. Sixty-two percent (248/401) of the women were referred to hepatology for further management of hepatitis B in pregnancy; 68% (48/70), 49% (44/89, of whom 20 women transferred care elsewhere and 1 miscarried), 86% (55/64) and 51% (101/200) were referred in H1, H2, H3 and H4 respectively.

**Figure 2:**
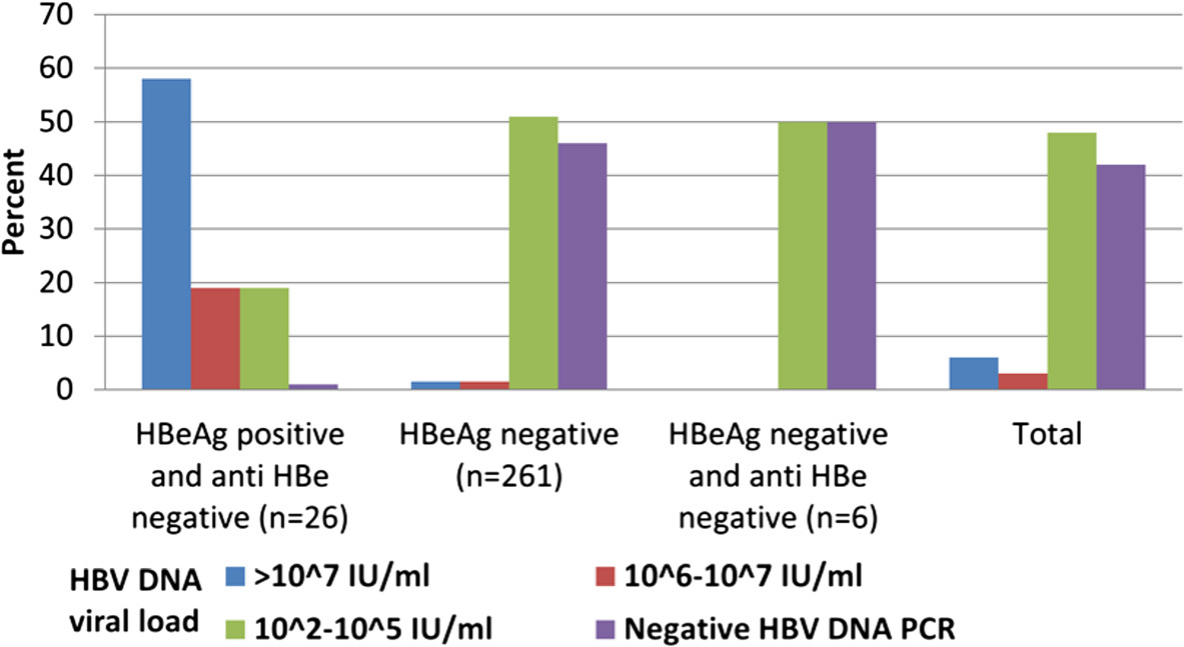
Patterns of hepatitis B viral load and serological markers among women in whom HBV DNA quantitation was undertaken in pregnancy (n = 261) in the 4 study hospitals over 2009–2010.

Overall, HBV DNA quantitation was carried out for 293 (73%) women: 76% (26/34) of those HBeAg positive and anti HBe negative, 73% (261/359) of those HBeAg negative and anti HBe positive and 75% (6/8) of those HBeAg negative and anti HBe negative. Patterns of VL stratified according to markers of infectivity are presented in Figure 2. Of the 293 women with VL quantitation, 18 (6%) had HBV DNA levels above 10^7^ IU/ml, 8 (3%) had levels at 10^6^–10^7^, 143 (49%) had levels of 10^2^–10^5^ and the remaining 124 (42%) had undetectable levels (Additional file 1: Table S1).

There was very high coverage of HIV testing, with 99% (399/401) women receiving an HIV antibody test. Approximately half (53%, 212/401) of the women received serological testing for hepatitis C and 31% (124/401) for delta virus. There was a wide variation in coverage of testing for delta virus between hospitals, ranging from 98% (H3) to 0% (H2), with coverage of 6% and 32% in H1 and H4 respectively. One woman (1/124, 0.8%) was positive anti-HDV and HDV RNA. Two hospitals tested around one-third of pregnant women with HBV infection for hepatitis C antibodies (H1 30% and H4 34%), compared with in excess of 80% in the other hospitals (H2 82% and H3 89%).

Antiviral treatment for pregnant women with hepatitis B was being offered in two of the study hospitals in 2009 and 2010. Of the 18 women with VL > 10^7^ IU/ml and thus eligible for antiviral therapy (Table 1), one had a miscarriage, 13 were under care at hospitals not providing antivirals and four were treated with antivirals (lamivudine or tenofovir) depending on local protocol. A further 9 women thought to be at high risk of transmitting hepatitis B to their infants (i.e. HBeAg positive, VL between 10^6^ to 10^7^ IU/ml, antiHBe and HBeAg negative) were also on antivirals.

### Management of infants

Of the 401 women, 6 miscarried and 69 transferred care to another hospital, giving 326 infants delivered in the audit hospitals, of whom all received the first dose of hepatitis B vaccine immediately after birth, with an intention to complete an accelerated course according to the Department of Health, UK guidelines (Table 3) [13]. There was no vaccination data available for the remaining 69 births to women who delivered elsewhere or were lost to follow up.

**Table 3:** Management of infants born to women with hepatitis B

**1**^**st **^**dose of hepatitis B vaccination**	**Hospital 1 n/70 (%)**	**Hospital 2 n/89 (%)**	**Hospital 3 n/64 (%)**	**Hospital 4 n/178 (%)**	**Total n/401 (%)**
Vaccinated	58 (83)	68 (76)	57 (89)	143 (80)	326 (81.3)
Miscarriages/deaths	1 (2)	1 (1)	1 (2)	3	6 (1.5)
No data	11 (15)	20 (22)	6 (11)	32 (18)	69 (17.2)
Total	70 (100)	89 (100)	64 (100)	178 (100)	401 (100)
**Hepatitis B immunoglobulin (HBIG) administration**	**Number of neonates given Hepatitis B immunoglobulin (HBIG) in addition to Hepatitis B vaccine**				
	**Hospital 1 (%)**	**Hospital 2 (%)**	**Hospital 3 (%)**	**Hospital 4 (%)**	**Total (%)**
HBIG given appropriately/number of high risk infants delivered in audit hospitals eligible for HBIG	11/11 (100)	2/7 (29)	5/8 (63)	18/22 (82)	36/48* (75)
HBIG given without indication	4	0	2	No data	6

*From Hospitals 2, 3 and 4: 2 women had a miscarriage and 10 moved care elsewhere.

The first dose of hepatitis B vaccine was administered by the maternity team, with a view of referring the baby to the general practitioner, community services or paediatricians to complete the remaining doses and check the anti HBs levels at the end of the primary course. Table 3 shows the number of infants of high-risk mothers who received HBIG in addition to vaccination. 48 infants were eligible for HBIG according to the national guidelines [13,14]; 36 infants received HBIG. Ten women with a high risk of transmission to the baby moved care or delivered elsewhere and a further two had a miscarriage. In addition to the 36 babies, it was found that 6 more babies from 2 hospitals received HBIG without an appropriate indication; this was attributed to different policies or guidelines being followed by hepatologists and paediatricians instead of following the national guidelines.

## Discussion

This multicentre retrospective audit, conducted in North Central London, was based on data of women who booked in the antenatal services at the four participating centres. The audit assessed adherence to the UK Department of Health guidelines, UK published in 2003 [13] for management of pregnant women with hepatitis B applicable at the time (i.e. 2009–2010). There is high coverage of antenatal screening for hepatitis B in the UK; in London, 98% of pregnant women were screened in 2006, with approximately 1200 infants born to mothers with HBV infection annually [17].

The prevalence of hepatitis B in pregnant women in our audit was found to be 1.05% of the total antenatal bookings. In England, in 2011 0.42% of pregnant women screened for HBV were HBsAg positive overall, with the highest prevalence in London at 1.02%; this represented a slight decline in overall prevalence over the past five years, from 0.47% in 2007 [18]. In a surveillance study of antenatal women in London, the overall prevalence of hepatitis B in 2007 was 1.17%, ranging from 0.26% to 2.39% across the NHS Trusts [19]. We demonstrated a wide variation in prevalence between hospitals, from 0.4% to 2.3%, which may have reflected differences in the local populations, particularly ethnicity. However our sector is a relatively high prevalence area.

Sixty percent of the pregnant women with hepatitis B were Black African or Asian, which is consistent with a recent study in Birmingham in which 33%, 24% and 11% of antenatal HBV cases in 2004–2008 were in Black African, Pakistani and Chinese women respectively; the highest prevalence of hepatitis B was in pregnant women of Black African (3.93%), White non-British (2.05%) and Pakistani (0.82%) origin [20]. However this was in contrast to a large cross sectional UK study which showed a predominance of South and South East Asian ethnicity amongst patients with hepatitis B [21].

The vast majority (data not shown) of infected women in the audit were newly diagnosed in pregnancy and not already under the care of hepatology services, thus representing an excellent opportunity for case detection and extended family testing. We found that referral of pregnant women with HBV to hepatology services was suboptimal, at only 62%. The NHS IDPS Programme standards for hepatitis B were updated in September 2010; these changes acknowledged the organisational challenges of screening and of appropriate maternal and infant management, which involves a range of teams and included key performance indicators, such as maternal referrals to be made to an appropriate specialist service for clinical assessment within 6 weeks of a confirmed positive HBsAg test [15]. The finding that two-fifths of infected women were not referred to specialist care underscores the need for this indicator. Standardisation of a patient referral pathway (Additional file 2: Figure S1) across the sector and strategies such as appointing a specialist midwife focusing on blood borne viruses/infections in pregnancy will facilitate referrals and help to achieve these targets. Future national audits will be able to investigate the impact that the updated standards have had on maternal management.

Although serological HBV markers were performed on all women diagnosed with HBV infection, HBV DNA quantitation was performed less frequently.

HBV DNA is a significant determinant of vertical transmission and the importance of viral load quantitation is illustrated by the case of the 3 women who would have been classified as “low risk” in the absence of HBV DNA levels. For the 27% of women in whom HBV DNA quantitation was not performed, risk of transmission could not be fully evaluated. National guidelines for HBV immunisation recommended that infants should receive HBIG where maternal HBV DNA exceeds 1 × 10^6^ IU/ml; the guidance states that viral load should not be quantified with the specific purpose of determining need for passive immunisation, but can be used for this purpose if measured for maternal health (Table 1). BVHG guidelines have recommended viral load measurement as standard in all people newly diagnosed with hepatitis B since 2008 [22]. With regard to the use of antivirals for transmission purposes, our findings reflect the audit period and the fact that the national guidance in 2003 did not encompass indications for antivirals or specialist referral pathways [13], although BVHG guidelines recommended that antivirals should be considered for women with high viral loads [22]. Only two of the four hospitals included in the audit provided antiviral therapy for preventing vertical transmission, with 4 women identified to be at high risk of transmission (HBV DNA >10^7^ IU/ml) commencing treatment, consistent with BVHG guidance [22].

Although not part of the screening programme, testing of patients with HBV infection for other blood borne viruses is good medical practice and should be routinely performed, as recommended by BVHG and international guidelines [22-24]. There was good coverage with antenatal HIV testing, as would be expected for a test offered routinely, and on an opt-out basis in the UK, but screening for hepatitis C and delta virus was carried out in substantially fewer women with HBV, with considerable variation between hospitals. Testing of other blood borne viruses should also be standardised and laboratory systems could aid in ensuring this is performed.

Overall, one in six women transferred care or were lost to follow-up prior to delivery, of which of particular concern was the proportion of women with a high risk of transmission (16%, 10/63). This highlights the importance of adopting policies and pathways focusing on this group of women and their infants, who represent a highly mobile population. All infants delivered in the audit hospitals were known to have received the first dose of hepatitis B vaccine. There was similarly good coverage with HBIG: of the mothers identified to be of high infectivity, 2 miscarried and 10 delivered elsewhere, with the infants of the remainder all receiving HBIG. In addition, we noted that a small number of infants (6) received HBIG despite not strictly meeting the high risk criteria. This was explained by conflicting guidelines being followed by paediatricians and hepatologists where maternal VL > 10^3^ IU/ml was used as a cut-off for administration of HBIG.

There were several limitations to this audit. There is lack of data about the exact gestational age of the women at the time of diagnosis of hepatitis B and absence of data on the number with hepatitis C or HIV co-infection. Data were not available for women who presented and were diagnosed with HBV infection in labour, and we were unable to assess the management of neonates delivered in other hospitals. Lack of complete data included in discharge summaries of women and neonates not only hampered the audit, but present considerable challenges (including further vaccination and contact tracing of families) to those responsible for the on-going care and management of the mother and infant, such as general practitioners, primary health care services, paediatricians and hepatologists. Audit of completion of the infant vaccination schedule was beyond the scope of this work. All the hospitals involved in the audit have since introduced new services and methods of working to improve the outcomes for women with hepatitis B and their infants (example of the improved patient referral pathway used by one hospital is shown in Additional file 2: Figure S1). These changes will be subject to a prospective audit to demonstrate their effectiveness.

## Conclusion

Standardisation of the pathway of management of women with hepatitis B and their infants based on national guidelines is required to improve the care provided across the sector, and rigorous auditing should be implemented. The new detection of maternal infection should be seized as an opportunity to provide appropriate care to the mother and family contacts as well as the child. Testing for other blood borne viruses should be included in the pathway. Data regarding the use of antivirals in pregnancy should be systematically collected as their use in pregnancy for reducing risk of transmission is relatively new and questions remain regarding efficacy and safety. As the management of these women and infants is complex, there is a need for the different teams within hospitals and the community to work much more closely together and improved intersectorial sharing of information is needed to reduce the risk of women of high infectivity being lost to follow up.

### Ethical approval

Ethical approval not required as this was an audit, however the audit was compliant with the local clinical governance policies at each participating hospital.

## Competing interests

The authors declare that they have no competing interests.

## Authors’ contributions

Conception and design of audit: EN. Data acquisition: GG, DI, TM, MB and AF. Analysis and interpretation of data: CT, GG, MB, TM, DI and AF. All authors contributed to drafting, revision and final approval of the final manuscript.

## Pre-publication history

The pre-publication history for this paper can be accessed here:

http://www.biomedcentral.com/1471-2393/13/222/prepub

## Supplementary Material

Additional file 1: Table S1 Hepatitis B serology and viral load in antenatal women with hepatitis B. Click here for file

Additional file 2: Figure S1 Example of referral pathway for antenatal women with HBV (HBsAg positive). Click here for file
