# Supplementary material for: Management of hepatitis B in pregnant women and infants: a multicentre audit from four London hospitals
Source: BMC Pregnancy Childbirth. 2013 Dec 1;13:222. doi: 10.1186/1471-2393-13-222 (PMC3879069; doi:10.1186/1471-2393-13-222)
Supplement: Additional file 1: Table S1 — Hepatitis B serology and viral load in antenatal women with hepatitis B. [file 1471-2393-13-222-S1.doc]

**Table S1:** Hepatitis B serology and viral load in antenatal women with hepatitis B

| HBV serology | HBV viral load (log IU/ml) | H1 | H2 | H3 | H4 | Total |
| --- | --- | --- | --- | --- | --- | --- |
| *HBeAg positive*  *Anti HBe negative* | >10^7^ IU/ml | 2 | 5 | 2 | 6 | 15 |
|  | 10^6^ - 10^7^ IU/ml | 3 | 0 | 2 | 0 | 5 |
|  | 10^2^ – 10^5^ IU/ml | 3 | 0 | 0 | 2 | 5 |
|  | negative | 0 | 0 | 1 | 0 | 1 |
|  | not measured | 0 | 2 * | 0 | 6 | 8 |
| *HBeAg negative* *anti HBe positive* | >10^7^ IU/ml | 1 | 0 | 0 | 2 | 3 |
|  | 10^6^ – 10^7^ IU/ml | 0 | 0 | 2 | 1 | 3 |
|  | 10^2^– 10^5^ IU/ml | 32 | 37 | 33 | 33 | 135 |
|  | negative | 27 | 31 | 23 | 39 | 120 |
|  | not measured | 0 | 14* | 0 | 84 | 98 |
| *antiHBe negative* *HBeAg negative* | >10^7^ IU/ml | 0 | 0 | 0 | 0 | 0 |
|  | 10^6^ - 10^7^ IU/ml | 0 | 0 | 0 | 0 | 0 |
|  | 10^2^ – 10^5^ IU/ml | 0 | 0 | 1 | 2 | 3 |
|  | negative | 2 | 0 | 0 | 1 | 3 |
|  | not measured | 0 | 0 | 0 | 2 | 2 |
| Total |  | 70 | 89 | 64 | 178 | 401 |

* patient moved away or transferred care to another hospital
