# Supplementary material for: Management of hepatitis B in pregnant women and infants: a multicentre audit from four London hospitals
Source: BMC Pregnancy Childbirth. 2013 Dec 1;13:222. doi: 10.1186/1471-2393-13-222 (PMC3879069; doi:10.1186/1471-2393-13-222)
Supplement: Additional file 2: Figure S1 — Example of referral pathway for antenatal women with HBV (HBsAg positive). [file 1471-2393-13-222-S2.ppt]

## Slide 1
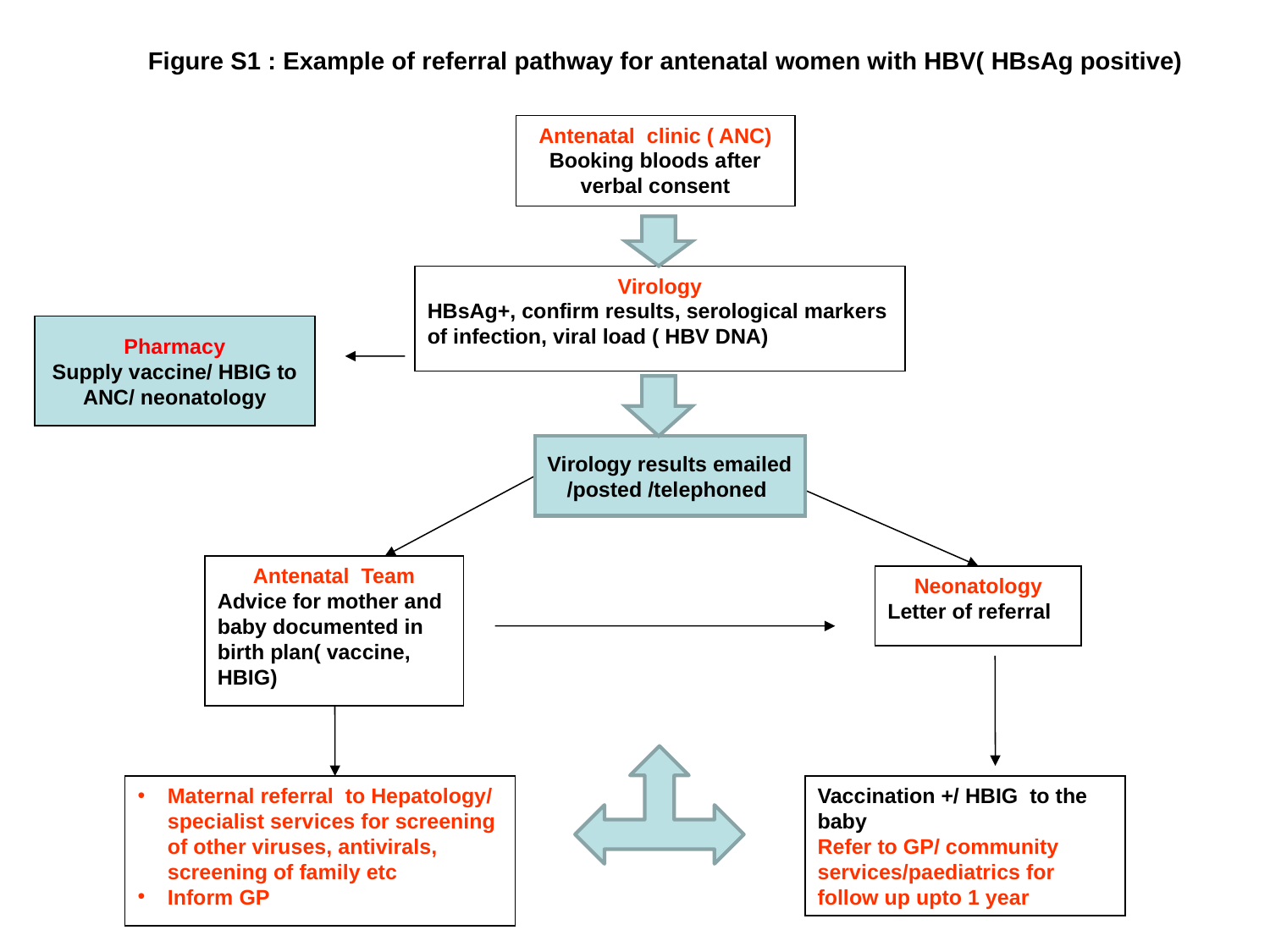

# Figure S1 : Example of referral pathway for antenatal women with HBV( HBsAg positive)
Antenatal clinic ( ANC)
Booking bloods after verbal consent
Virology
HBsAg+, confirm results, serological markers of infection, viral load ( HBV DNA)
Pharmacy
Supply vaccine/ HBIG to
ANC/ neonatology
Virology results emailed /posted /telephoned
Antenatal Team
Advice for mother and baby documented in birth plan( vaccine, HBIG)
Neonatology
Letter of referral
Maternal referral to Hepatology/ specialist services for screening of other viruses, antivirals, screening of family etc
Inform GP
Vaccination +/ HBIG to the baby
Refer to GP/ community services/paediatrics for follow up upto 1 year
